# Factors Affecting Nurses, Midwives and Allied Health Professionals' Ability to Engage With Research

**DOI:** 10.1111/jocn.70308

**Published:** 2026-03-29

**Authors:** Parveen Ali, Ahtisham Younas, Ashfaque Ahmed Talpur, Sam Debbage

**Affiliations:** ^1^ School of Allied Health Professions, Nursing and Midwifery University of Sheffield Sheffield UK; ^2^ Department of Education and Research, Doncaster and Bassetlaw Teaching Hospitals NHS Foundation Trust St John's Canada; ^3^ Faculty of Nursing Memorial University of Newfoundland Sheffield UK; ^4^ Director of Education and Research, Doncaster and Bassetlaw Teaching Hospitals NHS Foundation Trust Doncaster UK

**Keywords:** allied health professions, clinical care, healthcare research, midwifery, National Health Service, nursing, nursing practice, research capacity, research engagement

## Abstract

**Aim:**

To explore factors affecting research engagement among Nurses, Midwives and Allied Health Professionals (NMAHPs) in England by examining perceptions of research capacity at organisational, team and individual levels.

**Introduction:**

Research engagement strongly correlates with improved care quality. However, NMAHPs face persistent participation barriers compared to medical colleagues, limiting the development of a multi‐professional research workforce.

**Design:**

National descriptive cross‐sectional study using a validated survey tool.

**Methods:**

Data from NMAHPs across England were collected using the validated Research Capacity and Culture tool. Quantitative data were analysed descriptively and inferentially; qualitative free‐text responses were evaluated thematically.

**Results:**

Perceived capacity was moderate organisationally and in teams. Organisational strengths included promoting evidence‐based practice (68.7%) and leadership support (61.6%). Teams offered moderate research opportunities (58.6%) but limited mentorship (47.9%). Individually, participants showed competence in literature review (69.5%) and data collection (63.4%) but required support for funding acquisition (43.8%) and publication (50.0%). Qualitatively, research was a highly valued aspect of professional identity, though participation is severely constrained by structural conditions, including extreme resource pressures, unclear career pathways, and professional inequality.

**Conclusions:**

Despite strong motivation for evidence‐based practice, significant structural barriers restrict NMAHP research engagement. Strengthening capacity demands coordinated action across clinical and policy systems, ensuring equitable access to protected time, mentorship, and vital research infrastructure.

**Relevance for Clinical Practice:**

Supporting NMAHPs in research enhances evidence‐informed decisions and service innovation. Embedding research into everyday clinical work, rather than viewing it as optional, builds a sustainable multi‐professional culture.

**Impact:**

This survey pinpoints the specific factors most strongly influencing NMAHP research engagement. It provides healthcare leaders actionable insights to build sustainable research infrastructure and inclusive clinical academic pathways.

**Reporting Method:**

This study adhered to STROBE guidelines for cross‐sectional research.

**Patient or Public Contribution:**

No patient or public contribution.

## Key Highlights

- What Does This Paper Contribute to the Wider Global Clinical Community?
  ○ Provides large‐scale evidence on the organisational, team and individual factors influencing research engagement among Nurses, Midwives and Allied Health Professionals (NMAHPs), using data from a national sample across NHS settings.
  ○ Highlights persistent inequalities in access to mentorship, protected time, infrastructure, funding and career development, which can limit the development of clinical academic pathways for NMAHPs.
  ○ Offers practical insights to inform international strategies for strengthening inclusive, multi‐professional research cultures in clinical services, supporting the integration of evidence into everyday care.

## 1 Introduction

There is growing recognition that research engagement among clinicians is closely linked to enhanced organisational performance, improved clinical decision‐making, and better patient outcomes (Boaz et al. 2015; Jonker and Fisher 2018; Lee et al. 2024). For nurses in particular, involvement in research strengthens clinical judgement, encourages reflective practice, and supports a culture of organisational learning (Bradshaw 2001). Evidence suggests that nurse participation in research is associated with improved care quality and reductions in patient mortality (Boaz et al. 2015; Jonker and Fisher 2018). A strong clinical–academic interface enables nurses to evaluate existing practice, identify gaps in care, and implement evidence‐informed change. For example, Jarman et al. (2025) demonstrated that building research capacity among Nurses, Midwives and Allied Health Professionals (NMAHPs) increases meaningful involvement in research that directly informs clinical protocols. Nurses engaged in research, including multidisciplinary and collaborative research, are better equipped to respond to emerging demands in increasingly complex healthcare environments (NHS England 2023; Carrick‐Sen et al. 2019; Van Oostveen et al. 2017). Engaging with research early in a nursing career helps to develop critical thinking and evaluative skills, supporting the application of evidence to practice (Council of Deans of Health 2021). This expectation is also reflected within professional regulation, with the Nursing and Midwifery Council (NMC) emphasising that safe and effective practice must be grounded in research‐informed decision‐making (Nursing and Midwifery Council 2018).

The National Institute for Health and Care Research (National Institute for Health and Care Research 2023) further reinforces the central role of research engagement in professional development for NMAHPs. Their strategy highlights how research‐active environments contribute to staff retention, leadership development, and innovation in care delivery through the generation of new models of care, treatments, and technologies. However, unlike medicine and dentistry, where academic pathways are long‐established and embedded within professional training (Trusson et al. 2019; Bradshaw 2001), NMAHPs have historically had fewer opportunities to pursue clinical academic careers. Despite comprising the largest workforce group within the NHS, NMAHPs remain significantly under‐represented in research roles; fewer than 0.1% hold clinical academic posts, compared with 4.6% of medical consultants (Smith et al. 2018). In response, national policy aims to increase the proportion of NMAHP clinical academics to at least 1% by 2030 (Baltruks and Callaghan 2018). This ambition is now embedded within the UK research strategy, particularly through the 2024 Office for Strategic Coordination of Health Research (OSCHR) report, ‘Advancing healthcare through research’, which sets out a 10‐year vision to expand NMAHP clinical academic careers (OSCHR 2024).

To strengthen nursing and midwifery research leadership, the NIHR established the Senior Nurse and Midwife Research Leader (SNMRL; 70@70) programme in 2018, supporting senior clinicians to champion research engagement (National Institute for Health and Care Research 2019; Castro‐Sánchez et al. 2020). This forms part of a broader investment in clinical academic capacity‐building that includes pre‐master's research internships, pre‐ and post‐doctoral fellowships, and practical entry‐level initiatives such as the NIHR Associate Principal Investigator (PI) and Chief Investigator (CI) Scheme, developed to widen opportunities for NMAHPs to develop research leadership skills within clinical settings.

Targeted resources, including the NIHR Nursing Incubator and the Research Toolkit for Matrons, have also sought to normalise research leadership within nursing management (Henshall et al. 2020; NHS England 2025). The Health Education England Integrated Clinical Academic internship scheme has expanded access to early research training, although the long‐term impact of such initiatives is still developing (Nightingale et al. 2020). Despite this progress, persistent barriers continue to constrain research engagement among NMAHPs, including insufficient protected time, limited access to mentorship and training, service pressures, and a lack of organisational investment (Alexander et al. 2021; Avery et al. 2021; Bramley et al. 2018). For many nurses, balancing clinical workload with research aspiration remains challenging (Trusson et al. 2019; Lee et al. 2024).

Existing studies provide important insights but often focus on individual professional groups or single organisations or predate major national initiatives such as the SNMRL programme and ICA internship schemes (Castro‐Sánchez et al. 2020; Nightingale et al. 2020). Since the COVID‐19 pandemic, there has been renewed emphasis on embedding evidence‐based practice and strengthening research capacity to maintain service resilience (NHS England 2023). However, there remains limited contemporary, multi‐professional research examining NMAHP research capacity across organisational levels using validated approaches such as the Research Capacity and Culture (RCC) tool. The literature also continues to highlight gaps in understanding structural inequities, variation in research confidence, and access to mentorship across professions, all of which are essential to address to create inclusive and sustainable research pathways (Daniels et al. 2020).

This study contributes to this agenda by providing an up‐to‐date, large‐scale analysis of NMAHP research culture across England, focusing particularly on nurses' engagement in, and contribution to, multidisciplinary research. By combining quantitative and qualitative data, the study explores organisational, team‐level, and individual influences on research activity. The findings aim to inform national policy and local practice to ensure that nurses—alongside midwives and allied health professionals—are supported to participate in, lead, and shape research that improves patient care.

## 2 Study

The current study aimed to evaluate research capacity and culture among NMAHPs in England. We used the validated Research Capacity and Culture (RCC) tool to examine engagement across organisational, team, and individual levels. The study is guided by the following research questions:

- What are the perceptions of NMAHPS about research capacity at the organisational, team, and individual levels within the participating organisations?
- What are the barriers and motivators influencing NMAHPs in the participating organisations to engage in research?
- What are the perceptions of NMAHPs regarding their skill levels in research?

In this paper, ‘research engagement’ is defined broadly to encompass the full spectrum of research‐related activities, from using research evidence in practice to participating in, and ultimately leading, research projects. Engagement is not limited to formal clinical academic roles but reflects the wider research capacity and capability of the clinical workforce.

## 3 Methods/Methodology

### 3.1 Design

This study employed a descriptive cross‐sectional survey design to explore current perceptions of research capacity and engagement among NMAHPs across organisational, team and individual levels. The study followed the STROBE (Strengthening the Reporting of Observational Studies in Epidemiology) guidelines for cross‐sectional research.

### 3.2 Study Setting and Participants

The study sought to recruit registered NMAHPs working in NHS services in England, including Nurses, Midwives and Allied Health Professionals (AHPs). For this study, the AHP group included all 14 nationally recognised professions (e.g., Physiotherapists, Occupational Therapists, Radiographers). A full breakdown of participating professions is provided in Table 1. The term ‘NMAHP’ is used because it remains a recognised umbrella definition within UK health policy. We also adopted an inclusive approach by inviting participation from all registered non‐medical healthcare professionals, including pharmacists (See Table 1).

**TABLE 1:** Demographic characteristics of the participants (*n* = 644).

Characteristics	Nurses and Midwives (*n* = 349)	AHPs (*n* = 295)	Total	%
Age				
Under 20	17	6	23	3.6
20–29	58	55	113	17.5
30–39	84	98	182	28.3
40–49	88	81	169	26.2
50–60	89	50	139	21.6
Over 60	13	5	18	2.8
Gender				
Male	277	204	277	43.0
Female	68	88	68	10.6
Prefer not to say	4	3	4	0.6
Education				
Diploma	73		73	11.3
Degree (BSc, BA)	147	98	147	22.8
Masters	106	157	106	16.5
PhD/Doctorate	23	40	23	3.6
Professional experience				
Less than 1 year	22	14	36	5.6
1–5 years	63	80	143	22.2
6–10 years	39	54	93	14.4
Over 10 years	225	147	372	57.8
Profession				
Registered Nurses	256			39.8
Registered Midwife	50			7.8
Nursing Associate	43			6.7
Physiotherapist		62		9.6
Occupational therapist		35		5.4
Dietitian		32		5.0
Pharmacist		22		3.4
Chiropodist/Podiatrist		19		3.0
Radiographer		18		2.8
Psychologist		17		2.6
Clinical Scientist		16		2.5
Art therapist		12		1.9
Biomedical Scientist		11		1.7
Operating Department Practitioner		11		1.7
Paramedic		9		1.4
Speech and Language Therapist		9		1.4
Orthoptist/optometrist		9		1.4
Audiologist		6		0.9
Osteopath		4		0.6
Prosthetist/Orthotist		3		0.5

A convenience sampling approach was used to allow broad participation across multiple organisations. Recruitment was promoted via senior NMAHP leaders, professional networks, newsletters, targeted email distribution and social media platforms. This meant that it was not possible to determine the total number of staff who received the invitation. A formal response rate could therefore not be calculated. Given the descriptive and exploratory nature of the study, a formal a priori power calculation was not undertaken. The intention was to capture a large and diverse sample rather than test a specific hypothesis. However, the achieved sample of 644 respondents is comparable to, and larger than, previous RCC studies and is sufficient to support meaningful descriptive and exploratory comparative analysis.

### 3.3 Data Collection

Data were collected using an online questionnaire hosted on Google Forms, selected for accessibility and ease of use across devices and organisations. The survey was open for 3 months, from March 2024 to June 2024. The invitation link directed participants to a participant information sheet and consent statement before gaining access to the questionnaire. Completion and submission of the survey constituted informed consent. To preserve anonymity, no identifiable information was collected, and partial responses could not be saved for later completion. Where numbers were very small within a subgroup, responses were aggregated to protect confidentiality.

### 3.4 Instrument

We used the RCC tool (Holden et al. 2012), a 51‐item instrument, to assess perceptions of research capacity across three domains: Organisation level (18 items), team level (19 items), and individual level (14 items). Responses were recorded on a six‐point Likert scale (1 = no success/skill to 6 = highest success/skill). The tool has demonstrated excellent internal consistency across domains (*α* = 0.95 for organisation, 0.96 for team and 0.96 for individual levels). Additional closed‐ and open‐ended questions captured data on research involvement, perceived barriers and motivators, and demographic characteristics, including professional group, years in role and Agenda for Change (AfC) banding.

### 3.5 Data Analysis

Quantitative data were exported from Google Forms to Microsoft Excel and subsequently analysed using IBM SPSS Statistics (version 29). Descriptive statistics were calculated for each RCC domain, with frequencies, percentages, mean, and median values reported. Where subgroup numbers were small (< 5), data were aggregated to maintain anonymity. For example, the responses of Midwives and Nursing Associates were combined within the category of Nurses and Midwives (N&M), and all AHP groups have been combined (AHPs). For interpretive clarity, we considered mean scores of 1–2.49 as ‘low’, 2.5–3.49 as ‘moderate’, and 3.5–6 as ‘high’. To explore potential differences between professional groups, inferential analysis was undertaken using independent sample *t*‐tests to compare the mean scores for N&M and AHP for organisation, team, and individual level.

Qualitative data from open‐text responses (*n* = 289; only from NMAHPs) were analysed using Braun and Clarke's coding reliability thematic analysis approach (Braun and Clarke 2006, 2021). Two researchers independently coded an initial 20% of responses to develop a coding framework, which was then refined and applied to the full dataset. Codes were organised into potential themes, which were then reviewed against the coded extracts and the entire dataset. Themes were refined and named to capture the essence of the data they contained. Member checking was conducted with a subset of participants (*n* = 15) to verify theme authenticity. An audit trail documented analytical decisions. The research team included experienced nurses with expertise in research capacity development among NMAHPs. Reflexive discussions were held throughout analysis to consider how professional backgrounds and prior assumptions about research engagement might shape the interpretation of the data.

### 3.6 Ethical Considerations

Ethical approval was obtained from the Ethical Review Committee of the University of Sheffield. Consent was obtained electronically prior to participation, and anonymity was preserved throughout.

## 4 Findings

### 4.1 Demographical Data

The final sample consisted of 644 NMAHPs. Nurses and Midwives formed the largest group (*n* = 349, 50.5%), followed by AHPs (*n* = 295, 42.7%). A full breakdown of professional groups and roles is presented in Table 1. Among Nurses and Midwives, Registered Nurses formed the largest subgroup (*n* = 256), followed by Registered Midwives (*n* = 50) and Nursing Associates (*n* = 43). The AHP category encompassed a wide range of disciplines, with Physiotherapists (*n* = 62), Occupational Therapists (*n* = 35), and Dietitians (*n* = 32) being the most numerous.

The sample included predominantly experienced staff, with 57.7% reporting more than 10 years of professional experience. Participants were mainly aged 30–49 years, with lower representation among staff aged under 30 and over 60 years. Most participants were female (74.1%), compared to male participants (24.9%), with a small proportion (1%) preferring not to disclose their gender. Educational qualifications were generally high: over one‐third reported holding a master's degree, and approximately one in 10 reported a doctoral qualification. Respondents were represented across bands, with the majority in Band 6 and Band 7 roles (Table 1).

### 4.2 RCC Quantitative Results

Across the RCC domains, organisational‐level research capacity was rated lower than team‐level and individual‐level capacity. Participants reported stronger engagement with evidence use in practice; lower confidence in obtaining funding and ethics approvals, and limited access to protected time and formal research support.

#### 4.2.1 Perspective About Organisational Level Skills and Success

Participants reported moderate organisational success in developing a positive research culture (Table 2). The highest‐rated attribute was “Promotes clinical practice based on evidence” (N& M mean = 3.44; AHP mean = 3.34), indicating strong support for evidence‐based practice. Engagement with partners was also viewed positively, with “Engages external partners in research” (N&M mean = 3.03; AHP mean = 3.15) suggesting effective collaboration with academic and research institutions. In contrast, infrastructure‐ and resource‐related items received lower ratings. “Has software programmes for analysing research data” (N&M mean = 2.07; AHP mean = 2.17), “Has regular forums/bulletins to present research findings” (N&M mean = 2.53; AHP mean = 2.96), and “Has mechanisms to monitor research quality” (N&M mean = 2.52; AHP mean = 2.63) all fell within the low to moderate range, indicating limited availability of organisational resources to support research activity.

**TABLE 2:** Organisations skills and success.

Items	Nursing and Midwifery professionals (*n* = 349)				Allied health professionals (*n* = 295)			
	Mean	Median	IQR	Interpretation	Mean	Median	IQR	Interpretation
Has adequate resources to support staff research training	2.64	3	2	Moderate	2.72	3	2	Moderate
Has funds, equipment or admin to support research activities	2.55	3	2	Moderate	2.75	3	2	Moderate
Has a plan or policy for research development	2.83	3	2	Moderate	2.89	3	2	Moderate
Has senior managers that support research	3.00	3	2	Moderate	3.14	3	2	Moderate
Ensures staff career pathways are available in research	2.60	3	3	Moderate	2.71	3	2	Moderate
Ensures organisation planning is guided by evidence	2.93	3	2	Moderate	2.87	3	2	Moderate
Has service users involved in research	2.77	3	2	Moderate	2.96	3	2	Moderate
Accesses external funding for research	2.75	3	2	Moderate	2.92	3	2	Moderate
Promotes clinical practice based on evidence	3.44	4	2	Moderate	3.34	4	1	Moderate
Encourages research activities relevant to practice	2.86	3	2	Moderate	3.16	3	2	Moderate
Has software programs for analysing research data	2.07	2	3	Low	2.17	2	3	Low
Has mechanisms to monitor research quality	2.52	3	3	Moderate	2.63	3	3	Moderate
Has identified experts accessible for research advice	2.82	3	2	Moderate	3.06	3	2	Moderate
Supports a multi‐disciplinary approach to research	2.87	3	2	Moderate	3.02	3	2	Moderate
Has regular forums/bulletins to present research findings	2.53	3	3	Moderate	2.96	3	2	Moderate
Engages external partners (e.g., universities) in research	3.03	3	2	Moderate	3.15	3	2	Moderate
Supports applications for research scholarships/degrees	2.58	3	3	Moderate	2.93	3	2	Moderate
Supports the peer‐reviewed publication of research	2.69	3	3	Moderate	2.99	3	2	Moderate

“Unsure” responses were most frequent for items relating to data analysis software (25%), access to expert advice (20%), and mechanisms for monitoring research quality (20%), suggesting areas where awareness of available support may be limited.

Independent *T*‐test indicated a non‐statistically significant difference in the overall means for N&M (Mean = 2.74, SD 1.12) and AHPs (Mean = 2.90, SD 0.96) for the organisation domain (*t* (642) = −1.917, *p* = 0.056).

#### 4.2.2 Perspective About Team Level Skills and Success

Team‐level research capacity was generally rated lower than organisational‐level capacity across all professional groups. The highest‐rated team attributes were “Does planning that is guided by evidence” (N&M mean = 2.98; AHP mean = 3.06) and “Has team leaders that support research” (N&M mean = 2.94; AHP mean = 3.04), indicating that evidence‐based planning and collaborative working are valued within many teams (Table 3).

**TABLE 3:** Team level success and skills.

Items	Nursing and Midwifery professionals				Allied health professionals			
	Mean	Median	IQR	Interpretation	Mean	Median	IQR	Interpretation
Has adequate resources to support staff research training	2.54	3	3	Moderate	2.53	2	3	Moderate
Has funds, equipment or admin to support research activities	2.42	2	3	Low	2.46	2	3	Low
Does team level planning for research development	2.54	3	3	Moderate	2.62	3	3	Moderate
Ensures staff involvement in developing that plan	2.60	3	3	Moderate	2.68	3	3	Moderate
Has team leaders that support research	2.94	3	2	Moderate	3.04	3	3	Moderate
Provides opportunities to get involved in research	2.87	3	2	Moderate	2.94	3	3	Moderate
Does planning that is guided by evidence	2.98	3	2	Moderate	3.06	3	3	Moderate
Has service user involvement in research activities/planning	2.52	3	3	Moderate	2.63	3	3	Moderate
Has applied for external funding for research	2.42	2	3	Low	2.52	3	3	Moderate
Conducts research activities relevant to practice	2.90	3	2	Moderate	3.01	3	3	Moderate
Supports applications for research scholarships/degrees	2.51	3	3	Moderate	2.81	3	3	Moderate
Has mechanisms to monitor research quality	2.58	3	3	Moderate	2.55	3	3	Moderate
Has identified experts accessible for research advice	2.79	3	3	Moderate	2.96	3	3	Moderate
Disseminates research results at research forums/seminars	2.66	3	3	Moderate	2.85	3	3	Moderate
Supports a multi‐disciplinary approach to research	2.80	3	3	Moderate	3.10	3	3	Moderate
Has incentives & support for mentoring activities	2.38	2	3	Moderate	2.36	2	3	Moderate
Has external partners (e.g., universities) engaged in research	2.70	3	3	Moderate	2.92	3	3	Moderate
Supports peer‐reviewed publication of research	2.55	2	3	Moderate	2.88	3	3	Moderate
Has software available to support research activities	2.18	2	2	Low	2.34	2	3	Low

However, substantial limitations were noted in relation to research infrastructure. The lowest ratings were reported for “Has software available to support research activities” (N&M mean = 2.18; AHP mean = 2.34) and “Has funds, equipment or admin to support research activities” (N&M mean = 2.42; AHP mean = 2.46).

Uncertainty was also evident for some items. For example, 17.2% of participants were unsure about accessing external research funding, and more than 14% were unsure about identifying experts for research advice or mechanisms for monitoring quality. The highest uncertainty (16.8%) related to the availability of software to support research activities, suggesting that awareness of these supports may be limited.

Independent *T*‐test indicated a non‐statistically significant difference in the overall means for N&M (Mean = 2.62, SD 1.30) and AHPs (Mean = 2.75, SD 1.12) for the team domain (*t* (642) = −1.291, *p* = 0.197).

#### 4.2.3 Perspectives About Individual Level Skills and Success

The data indicate a consistent pattern in individual research capabilities, with strengths in foundational research skills and lower confidence in more advanced activities. “Finding relevant literature” received the highest rating (mean = 3.58), with both AHPs and Nurses and Midwives (N&M) rating this as high (median = 4). “Critically reviewing the literature” (mean = 3.39) and *“Collecting data”* (mean = 3.36) were also rated favourably, suggesting a broadly positive level of engagement with basic research activities across professional groups.

The lowest‐rated individual skill across all professional groups was “Securing research funding” (mean = 2.42), with both AHPs and N&M rating this as low (median = 2.00). Lower ratings were also reported for “Submitting an ethics application” and “Writing a research report”, indicating limited confidence in advanced research tasks.

Statistical analysis showed significant differences between AHPs and N&M in several domains after Bonferroni correction. AHPs reported significantly higher skill levels in “Writing a research protocol” (mean 3.00 vs. 2.58, *p* < 0.001), “Submitting an ethics application” (mean 2.88 vs. 2.50, *p* = 0.003), and “Writing a research report” (mean 3.16 vs. 2.76, *p* = 0.003), as shown in Table 4. “Unsure” responses were slightly more frequent for items relating to securing research funding and submitting ethics applications, which were also the skills showing group differences. For this domain, independent *t*‐test indicated statistically significant difference in the overall means for N&M (Mean = 2.66, SD 1.10) and AHPs (Mean = 2.95, SD 1.07) (*t* (642) = −3.397, *p* = < 0.001). Cohen's d indicated a small effect size (*d* ≈ 0.27), suggesting modest practical differences between professional groups.

**TABLE 4:** Individual level success and skills.

Items	Nursing and Midwifery professionals				Allied health professionals			
	Mean	Median	IQR	Interpretation	Mean	Median	IQR	Interpretation
Finding relevant literature	3.60	4	1	High	3.54	4	1	High
Critically reviewing the literature	3.35	3	1	Moderate	3.40	3	1	Moderate
Using a computer referencing system (e.g., Endnote)	3.01	3	2	Moderate	3.09	3	2	Moderate
Writing a research protocol	2.39	2	2	Low	2.86	3	2	Moderate
Securing research funding	1.99	2	2	Low	2.47	2	3	Low
Submitting an ethics application	2.26	2	2	Low	2.72	3	3	Moderate
Designing questionnaires	2.78	3	2	Moderate	3.07	3	2	Moderate
Collecting data e.g., surveys, interviews	3.11	3	2	Moderate	3.24	3	2	Moderate
Using computer data management systems	2.73	3	3	Moderate	2.89	3	2	Moderate
Analysing qualitative research data	2.60	3	2	Moderate	2.90	3	2	Moderate
Analysing quantitative research data	2.50	3	3	Moderate	2.87	3	2	Moderate
Writing a research report	2.54	3	3	Moderate	3.01	3	2	Moderate
Writing for publication in peer‐reviewed journals	2.35	2	3	Low	2.71	3	3	Moderate
Providing advice to less experienced researchers	2.58	3	3	Moderate	2.78	3	3	Moderate

### 4.3 Themes From Qualitative Data

Analysis of the open‐text responses generated from 289 responses resulted in the development of two overarching themes: Research as a valued aspect of professional identity and practice, and (2) Structural conditions constraining research engagement. The first theme reflects contexts in which research is embedded within professional culture and identity. The second theme captures multi‐level constraints perceived to limit NMAHP access to, and participation in, research.

#### 4.3.1 Research as a Valued Aspect of Professional Identity and Practice

Participants described research involvement as contributing positively to their sense of professional identity, learning and team culture. Where research activity was visible and supported, it was perceived not simply as an additional task but as part of “how we work”.

Several respondents highlighted the professional satisfaction and collaborative connections that research enabled, particularly within the multi‐disciplinary team (MDT):

Research has provided the opportunity to work together as an MDT to present and share findings with international partners, which has been very rewarding. For example, meeting international colleagues, setting up international nursing groups and studies, and presenting at international conferences. … research has changed nursing practice in our team to improve patient care. (Nurse 12)

For others, research involvement generated motivation, pride and enthusiasm within their teams:

Our team is led by a very strong research ethic; therefore, it feels like we are encouraged to get involved in research and this is motivating… Some team members undertaking master's level qualifications are doing research projects… beginning to push their enthusiasm for research feeding into practice. (Nurse 04)

Intellectual curiosity and the desire to use research to answer clinical questions were also strongly expressed:

… curiosity, desire to base practice on evidence… something different than just clinical practice and an ability to find the answers to the clinical questions. (AHP 23)

Together, these accounts illustrate that, where opportunities and support exist, research engagement is experienced by NMAHPs as meaningful, confidence‐building and integral to practice rather than peripheral to clinical work.

#### 4.3.2 Structural Conditions Constraining Research Engagement

Alongside positive accounts, participants also described research engagement as constrained by a set of structural, organisational, and professional factors. These influences operated across leadership, resources, opportunities and pathways, communication, and professional status.

##### 4.3.2.1 Leadership and Management Support

Many respondents perceived limited or inconsistent support for research from senior management. Some reported being discouraged from pursuing research careers, or that research activity was insufficiently recognised:

It is much harder to get the in‐house support you need to engage in research and pursue a research career. I have at times been actively discouraged from pursuing a research career by senior management as it would not make a (positive) difference to my nursing career. (Nurse 52)

Several participants felt that operational service delivery was prioritised above reflective, evidence‐generating practice:

Senior management or clinical managers are focused on providing direct patient care and delivering services more than taking a step back and looking to see if what we are doing is the best evidence or if more evidence is required. (Nurse 71)

Lack of recognition for research contributions was also described:

Poor leadership focuses on clinical and not recognition of what I do or have achieved (Nurse 34).

##### 4.3.2.2 Resource Constraints

Participants consistently reported that limited time, funding, backfill, and infrastructure restricted their ability to engage in research:

Often, nursing staff are interested in research, but there is insufficient support or funding to backfill. Research nurse roles and the CRF (Clinical Research Facility) management structure are the only viable approaches to fund research. Research developed by nurses, where the nurse assumes the role of Principal Investigator, is lacking as a result. (Nurse 17)

Building on this, participants also reported a broader organisational issue of adding additional responsibilities without the corresponding resources or support, as one participant succinctly stated:

The time and headspace needed to undertake any stage of research is vastly underestimated and remains a significant barrier. Moving forward often necessitates completing a substantial portion of the required research work in your own time. (Midwife 13)

##### 4.3.2.3 Opportunities and Pathways

Respondents reported a lack of clear education, training and career pathways into research roles, particularly for early‐career staff:

I was supported by the trust to do the NIHR MSc in clinical research and then do some research secondment. However, once I secured my PhD, they would not support this. I had to leave and now hold a zero‐hours contract only. There are no clinical academic roles/opportunities with therapy services. (Nurse 80)

Uncertainty surrounding career progression was frequently noted:

No clear research career pathway laid out for all professions. (AHP 34)

…no opportunities for band 5 nurses to complete PhD study and… little support or funding offered towards career development. (Nurse 20)

##### 4.3.2.4 Communication Gaps in NMAHP Research Communities

Several participants reported limited awareness of research opportunities or uncertainty about how to access them:

We are not typically informed of what research is happening, and for those of us that are interested in research, it is unclear how to access this… access to this during a workday is unrealistic. (Nurse 108)

Multiple participants also repeatedly indicated insufficient communication about research opportunities within their trusts, which has led to a lack of awareness of any potential opportunities to engage in research. This theme reiterates the importance of clear, active communication to improve collaboration and promote research opportunities.

##### 4.3.2.5 Professional/Structural Inequality

Several respondents perceived inequity between medical staff and NMAHPs in access to research opportunities, resources and recognition:

My organisation is a strong place to do research as a medic so the answers regarding research readiness are completely different if you are a nurse… or a medic doing research. (Nurse 10)

…this is not aimed at the multi‐disciplinary team as a whole but specifically at doctors as part of their training and engagement. They go into research, but other staff are neither encouraged nor given many opportunities. (AHP 19)

Some participants felt that nursing and AHP research contributions were undervalued:

…research is not really part of the nursing world, and only doctors get access to research. (Nurse 20)

These accounts highlight perceptions of persistent professional hierarchies that shape who is seen and supported as a legitimate research contributor.

## 5 Discussion

This study offers a comprehensive and contemporary examination of research capacity and culture among NMAHPs in the UK. Using a cross‐sectional survey incorporating both quantitative and qualitative data, it explored perceptions of research capacity at organisational, team and individual levels, alongside qualitative insights into the structural and cultural conditions shaping research engagement. While many of the barriers reported here are familiar from earlier work, the contribution of this study lies in its scale, post‐pandemic timing, multi‐professional scope, and integration of quantitative and qualitative findings. The Evidence‐to‐Practice Matrix synthesises these findings into an actionable framework, linking barriers with practical responses across system levels.

The sample comprised predominantly experienced clinicians, with over half reporting 10 years' experience and high levels of postgraduate education. This profile suggests a workforce with substantial clinical expertise and a strong foundation from which to build research capability. However, early‐career and lower‐band staff were under‐represented, echoing concerns that research engagement opportunities remain more accessible later in professional careers (Trusson et al. 2019). These risks reinforce inequitable access to research careers and may limit the development of sustainable research pipelines.

At the organisational level, respondents reported moderate success in fostering research‐positive environments, particularly in relation to evidence‐based practice and partnerships with external research organisations. These findings align with earlier work highlighting the importance of leadership commitment and institutional support in enabling NMAHP‐led research (Carrick‐Sen et al. 2019; Jarman et al. 2025). Yet, persistent deficits were identified in protected time, administrative support, funding access and research infrastructure. Difficulty navigating governance processes and securing research funding were reported as major deterrents, reflecting longstanding structural inequities in how research funding has historically been distributed between medical and non‐medical professions (Jones and Keenan 2021; Lee et al. 2024).

Team‐level findings mirrored organisational‐level trends. Respondents valued teams that encouraged evidence use and multi‐professional collaboration, yet many described insufficient local mentoring and limited access to external funding. Qualitative data reinforced these findings, with participants frequently reporting staffing pressures, workload intensity and lack of backfill as barriers, themes widely reported in AHP and Nursing & Midwifery research literature internationally (Pager et al. 2012; Cordrey et al. 2022; Lee et al. 2024). The perceived reluctance or lack of capacity among middle managers to support research engagement remains a critical constraint (Carrick‐Sen et al. 2019; Richardson et al. 2019).

Across professions, respondents expressed confidence in foundational research skills, such as literature searching and critical appraisal. However, confidence declined sharply in relation to advanced research tasks, including protocol development, ethics applications and, most markedly, securing funding. These findings are consistent with previous national and international literature (Matus et al. 2020; Palmer et al. 2023) and highlight enduring system‐level capability gaps.

Quantitative results showed that AHPs reported significantly higher confidence than Nurses and Midwives in several advanced research activities. This may reflect differences in pre‐ and post‐registration education pathways, in which research components can be more formally embedded in some AHP programmes (Trusson et al. 2019). However, our qualitative analysis suggests that these skills are often developed despite, rather than because of, workplace research infrastructure, pointing to inequities in access to institutional research development opportunities.

Where research engagement was enabled, participants described it as an integral part of professional identity, team culture, and high‐quality care. Research was associated with motivation, pride, intellectual curiosity and belonging within the MDT. However, participants also described structural conditions that restrict research engagement, including inconsistent leadership support, inadequate resourcing, unclear career pathways, weak communication systems and entrenched professional hierarchies. Many perceived that research careers remain culturally aligned with medicine, while NMAHP roles are structurally positioned around service delivery. These themes echo national reports calling for systemic expansion of NMAHP clinical academic career pathways (Baltruks and Callaghan 2018; OSCHR 2024).

Importantly, these structural constraints do not reflect lack of motivation or capability; rather, they shape who is seen as a legitimate research contributor and who must undertake research in their own time.

Taken together, the findings demonstrate that individual capability is present, but system‐level conditions remain insufficiently supportive. This pattern suggests a misalignment between individual research capability and the organisational and structural conditions intended to support research engagement. While participants reported confidence in foundational research skills and a strong professional commitment to evidence‐informed practice, many described limited access to protected time, infrastructure and clear career pathways. These findings indicate that research engagement is shaped not only by individual motivation or competence, but by wider organisational and professional contexts that influence how research roles are legitimised and supported within health systems. Meaningful research engagement, therefore, requires coordinated action across organisational, team and professional levels, not isolated training initiatives (Cowley et al. 2020). The Evidence‐to‐Practice Matrix operationalises this by mapping barriers to specific actions.

The qualitative data also remind us that progress is constrained by wider structural pressures—including workforce shortages, budgetary constraints and models of service delivery that prioritise immediate clinical throughput over long‐term research investment. Dedicated research time in job plans remains widely advocated but inconsistently implemented (Baltruks and Callaghan 2018). Developing equitable clinical academic pathways requires collaboration between NHS organisations, HEIs, funders and regulators, supported by strong middle‐manager engagement and local cultural change (Cowley et al. 2020).

This study demonstrates that NMAHPs value research and possess strong foundational capability but continue to experience structural inequity in research opportunity and support. Strengthening NMAHP research engagement is therefore not simply a training issue, but a workforce, cultural and policy challenge.

### 5.1 Strengths and Limitations

This study has several important strengths. The large and professionally diverse sample provides a strong basis for comparing perceived research capacity across NMAHP disciplines and NHS settings. Use of the validated RCC tool enabled systematic assessment at organisational, team and individual levels, offering a comprehensive multi‐level perspective that is rarely captured in single studies. The inclusion of both quantitative and qualitative data further strengthened the work by combining quantitative assessment with qualitative insights, allowing exploration of not only the extent of research capacity but also the contextual and structural factors shaping NMAHPs' opportunities to engage in research.

However, several limitations must be acknowledged. The use of convenience and snowball sampling meant that a response rate could not be calculated, and the sample may over‐represent those already interested in research. This may have resulted in an overestimation of perceived research capacity, particularly at the individual level. The cross‐sectional design provides only a single time‐point snapshot and cannot capture how research capacity develops or responds to organisational or policy change. Early‐career staff were under‐represented, limiting insight into the experiences of those at the start of the career pipeline.

The RCC tool measures self‐reported perceptions rather than objective competence or research output, and responses may be influenced by confidence, professional identity, or exposure to research‐active environments. We were also unable to distinguish between respondents working in purely clinical roles and those in joint clinical‐academic or research‐focused posts, who may face different barriers and enabling factors. In addition, organisational identifiers were not collected, preventing analysis of variation across settings such as community, district general, and teaching hospital environments. Finally, the study is situated within the UK healthcare, funding and regulatory context. While many findings are likely to be transferable, caution is required when applying them to health systems with different workforce and research structures.

### 5.2 Implications for Clinical Practice, Policy and Research

The findings highlight a clear need to strengthen research engagement for NMAHPs within everyday clinical settings. Research participation is most feasible where leadership actively endorses it, workloads are realistic, and staff have equitable access to resources, mentorship and support. Protected time, administrative assistance and access to appropriate software and data systems are particularly important practical enablers. Targeted development in areas such as research design, ethics applications and grant writing may help address the lower confidence reported in advanced research skills, while structured mentorship is likely to be especially valuable for early‐career practitioners. Embedding research awareness and skills within quality improvement activity may also help integrate research into routine care rather than viewing it as an additional burden. Overall, the findings support the development of inclusive approaches to research engagement that recognise the diversity of NMAHP roles and contexts.

From a policy perspective, this study reinforces the importance of creating sustainable clinical academic pathways for NMAHPs. Despite national strategic commitments, access to research careers and funding opportunities remains uneven, with existing systems still more strongly aligned with medical research careers (Baltruks and Callaghan 2018; OSCHR 2024). If the UK is to realise the ambition of a genuinely multi‐professional research workforce, policy must support protected research time, streamlined research governance processes, fair access to funding, and strong partnership working between NHS organisations and universities. Recognising research contribution explicitly within job planning and appraisal systems would further strengthen its legitimacy as part of clinical work, rather than an optional add‐on.

Future research should examine the effectiveness of interventions such as protected research time, structured mentorship and clinical academic pathways in improving research participation, career progression and ultimately patient care outcomes. Longitudinal and mixed‐methods designs would be particularly valuable in examining how research engagement develops across career stages. Further work is also needed to understand inequalities in research opportunity—including differences by profession, gender, career stage and organisational context to ensure that new research initiatives do not inadvertently reinforce existing hierarchies. Evaluations of organisational culture and leadership development initiatives would also help clarify how supportive environments for NMAHP‐led research can be sustained. The following table (Table 5) maps key findings to practical actions for leaders, managers, educators and NMAHPs, providing a simple framework to guide strengthening of research engagement within clinical services.

**TABLE 5:** Evidence‐to‐practice matrix: translating research engagement into clinical action.

Study finding	Source	Clinical implication	Suggested action
Limited protected time and variable leadership support	Quantitative & qualitative	Reduced innovation and inconsistent engagement	Incorporate research time and support into job planning and appraisal systems
Unequal access to mentorship and career progression	Mainly quantitative	Slower skill development and retention challenges	Develop structured mentorship schemes and clinical academic pathways
Strong capability in literature searching and data collection	Quantitative	High readiness for evidence‐informed practice	Embed research skills within service improvement and audit activities
Communication gaps and lack of awareness of opportunities	Mainly qualitative	Missed opportunities for staff development	Establish accessible research communication hubs and clear organisational points of contact

## 6 Conclusion

This study provides a contemporary, multi‐level account of how NMAHPs perceive research capacity and culture within the UK NHS. Despite sustained national efforts to strengthen clinical academic pathways, many of the barriers to research engagement identified over the past decade remain evident, particularly in relation to protected time, mentorship, infrastructure and access to research opportunities. The findings suggest that research capability is most likely to flourish where research is legitimised within everyday clinical work and where staff have equitable access to structured support and development. Strengthening NMAHP research engagement offers an important route to advancing evidence‐based practice, service innovation and workforce development. However, progress will depend on coordinated action across clinical, organisational and policy systems, alongside attention to equity and access. Future initiatives should explicitly connect research engagement with patient care, ensuring that clinical academic development is embedded, supported and sustainable across the NMAHP workforce.

## Funding

The authors have nothing to report.

## Conflicts of Interest

The authors declare no conflicts of interest.

## Supporting information


**Data S1:** jocn70308‐sup‐0001‐supinfo.docx.

## Data Availability

The data that support the findings of this study are available on request from the corresponding author. The data are not publicly available due to privacy or ethical restrictions.
